# Genome sequence of the moderately halophilic bacterium *Salinicoccus carnicancri* type strain Crm^T^ (= DSM 23852^T^)

**DOI:** 10.4056/sigs.3967649

**Published:** 2013-06-03

**Authors:** Dong-Wook Hyun, Tae Woong Whon, Yong-Joon Cho, Jongsik Chun, Min-Soo Kim, Mi-Ja Jung, Na-Ri Shin, Joon-Yong Kim, Pil Soo Kim, Ji-Hyun Yun, Jina Lee, Sei Joon Oh, Jin-Woo Bae

**Affiliations:** 1 Department of Life and Nanopharmaceutical Sciences and Department of Biology, Kyung Hee University, Republic of Korea; 2 ChunLab, Inc., Seoul National University, Seoul, Republic of Korea

**Keywords:** moderately halophilic, *Salinicoccus carnicancri*, *Staphylococcaceae*

## Abstract

*Salinicoccus carnicancri* Jung *et al*. 2010 belongs to the genus *Salinicoccus* in the family *Staphylococcaceae*. Members of the *Salinicoccus* are moderately halophilic and originate from various salty environments. The halophilic features of the *Salinicoccus* suggest their possible uses in biotechnological applications, such as biodegradation and fermented food production. However, the genus *Salinicoccus* is poorly characterized at the genome level, despite its potential importance. This study presents the draft genome sequence of *S. carnicancri* strain Crm^T^ and its annotation. The 2,673,309 base pair genome contained 2,700 protein-coding genes and 78 RNA genes with an average G+C content of 47.93 mol%. It was notable that the strain carried 72 predicted genes associated with osmoregulation, which suggests the presence of beneficial functions that facilitate growth in high-salt environments.

## Introduction

The genus *Salinicoccus* in the family *Staphylococcaceae* was first proposed by Ventosa *et al*. (1990) and is defined as moderately halophilic, aerobic, Gram-positive, non-motile, non-sporulating, and heterotrophic cocci [1]. The genus name is derived from the Latin adjective *salinus,* saline, and the Greek masculine noun *kokkos*, meaning a grain or berry, i.e., saline coccus [2]. Most species in the genus *Salinicoccus* have been found in salty environments, such as fermented foods [3-5], solar salterns [1,6], salt mines [7,8], a salt lake [9], and saline soils [10,11]. All type strains of *Salinicoccus* species were characterized as halotolerant organisms, where NaCl concentrations of 2–20% (wt/vol) were suitable for growth [12-14].

These moderately halophilic bacteria can survive in salt-rich environments and grow optimally at 5–20% (wt/vol) NaCl [15]. These bacteria can utilize compatible solutes or osmolytes, such as carbohydrates, amino acid, polyols, betaines, and ectoines, by regulating their osmotic concentrations in high-salt content environmental conditions [16,17]. Therefore, these organisms may have biotechnological importance with possible applications in food biotechnology for the production of fermented food [18], in environmental biotechnology for the biodegradation of organic pollutants and the production of alternative energy [19].

Strain Crm^T^ (= DSM 23852 = JCM 15796 = KCTC 13301) is the type strain of the species *Salinicoccus carnicancri*. This strain was isolated from a traditional Korean fermented seafood, known as ‘ganjang-gejang,’ which is made from raw crabs preserved in soy sauce [20]. The species name was derived from the Latin nouns *caro carnis*, flesh, and *cancer -cri,* a crab, i.e., the flesh of a crab [2]. The strain can grow in 0–20% (wt/vol) NaCl with optimal growth at 12% (wt/vol) NaCl [20]. The present study summarizes the features of *S. carnicancri* strain Crm^T^ and provides an analysis of its draft genome sequence, which is the first reported genome sequence of a species in the genus *Salinicoccus*.

## Classification and features

**Figure 1:**
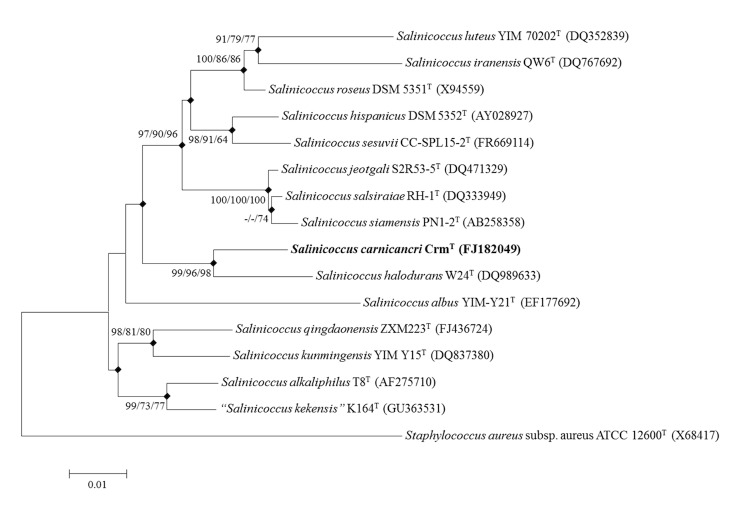
Phylogenetic consensus tree based on 16S rRNA gene sequences showing the relationship between *Salinicoccus carnicancri* strain Crm^T^ and the type strains of other species in the genus *Salinicoccus*. The type strain of *Staphylococcus aureus* was used as an outgroup. The GenBank accession numbers for the 16S rRNA genes of each strain are shown in parentheses. Filled diamonds indicate identical branches present in the phylogenetic consensus trees constructed using the neighbor-joining (NJ), maximum-parsimony (MP), and maximum-likelihood (ML) algorithms. The numbers at the nodes represent the bootstrap values as percentages of 1,000 replicates and values <70% are not shown at the branch points. The scale bar represents 0.01 nucleotide change per nucleotide position.

A taxonomic analysis was conducted based on the 16S rRNA gene sequence. The representative 16S rRNA gene sequence of strain *S. carnicancri* Crm^T^ was compared with the most recent release of the EzTaxon-e database [21]. The multiple sequence alignment program CLUSTAL W [22] was used to generate alignments with other gene sequences collected from databases. The alignments were trimmed and converted to the MEGA format before phylogenetic analysis. Phylogenetic consensus trees were constructed based on the aligned gene sequences using the neighbor-joining [23], maximum-parsimony [24], and maximum-likelihood [25] methods with 1,000 randomly selected bootstrap replicates using MEGA version 5 [26]. The phylogenetic analysis based on the 16S rRNA gene sequence showed that strain Crm^T^ was most closely related to *Salinicoccus halodurans* W24^T^ with 96.99% similarity. The phylogenetic consensus tree based on the 16S rRNA gene sequences indicated that strain Crm^T^ was clustered within a branch containing other species in the genus *Salinicoccus* (Figure 1).

Strain Crm^T^ (Table 1) was isolated from the fermented seafood ganjang-gejang during a project that investigated microbial communities in fermented foods, i.e., the Next-Generation BioGreen 21 Program (No. PJ008208) in Korea. Ganjang-gejang, with a NaCl (w/v) concentration of 24.5%, was produced by preserving scabbard crabs in soy sauce, garlic, and onions at –5°C for 4–5 days.

**Table 1:** Classification and general features of *Salinicoccus carnicancri* strain Crm^T^ according to the MIGS recommendations [27].

**MIGS ID**	**Property**	**Term**	**Evidence code**
		Domain *Bacteria*	TAS [28]
		Phylum *Firmicutes*	TAS [29-31]
		Class *Bacilli*	TAS [32,33]
	Current classification	Order *Bacillales*	TAS [34,35]
		Family *Staphylococcaceae*	TAS [36,37]
		Genus *Salinicoccus*	TAS [1,38]
		Species *Salinicoccus carnicancri*	TAS [20]
		Type strain Crm^T^	TAS [20]
	Gram stain	Positive	TAS [20]
	Cell shape	Cocci	TAS [20]
	Motility	Non-motile	TAS [20]
	Sporulation	Non-sporulating	TAS [20]
	Temperature range	4–45°C	TAS [20]
	Optimum temperature	30–37°C	TAS [20]
	Salinity range	0–20% (w/v)	TAS [20]
	Optimum salinity	12% (w/v)	TAS [20]
	pH range	6–11	TAS [20]
	Optimum pH	7–8	TAS [20]
	Carbon source	Heterotroph	TAS [20]
	Energy source	Not reported	
MIGS-6	Habitat	Fermented seafood (marinated crab)	TAS [20]
MIGS-6.1	Temperature	–5 to 5°C	IDA
MIGS-6.3	Salinity	20%	IDA
MIGS-22	Oxygen	Aerobic	TAS [20]
MIGS-15	Biotic relationship	Free-living	TAS [20]
MIGS-14	Pathogenicity	Unknown	
	Biosafety level	1	
MIGS-23.1	Isolation	The traditional Korean fermented seafood ‘ganjang-gejang’ (Crabs preserved in soy sauce)	TAS [20]
MIGS-4	Geographic location	Republic of Korea	TAS [20]
MIGS-5	Sample collection time	August, 2010	NAS
MIGS-4.1	Latitude	Not reported	
MIGS-4.2	Longitude	Not reported	
MIGS-4.3	Depth	Not reported	
MIGS-4.4	Altitude	Not reported	

Evidence codes, as follows: IDA: inferred from direct assay; TAS: traceable author statement (i.e., a direct report exists in the literature); NAS: non-traceable author statement (i.e., not observed directly in a living, isolated sample, but based on a generally accepted property of the species, or anecdotal evidence). These evidence codes are from the Gene Ontology project [39].

**Figure 2:**
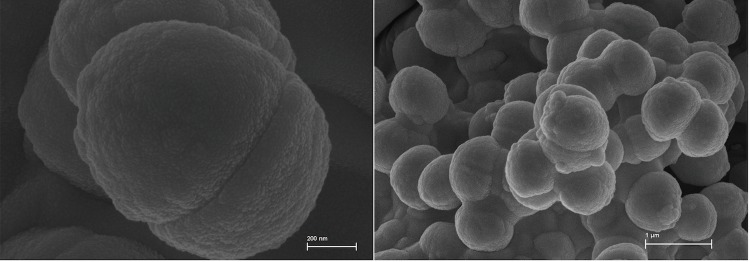
Scanning electron microscopy images of *S. carnicancri* Crm^T^ obtained using a SUPRA VP55 (Carl Zeiss) at an operating voltage of 15kV. The scale bars represents 200 nm (left) and 1 μm (right), respectively.

*S. carnicancri* strain Crm^T^ is a Gram-positive, moderately halophilic, non-motile, non-sporulating, and aerobic heterotrophic coccus with a diameter of 1.0–2.5 μm [20]. Figure 2 shows the morphological features of strain Crm^T^, which were obtained by scanning electron microscopy (SEM). Colonies were ivory-colored [20]. Growth occurred at 4–45°C, with an optimum of 30–37°C, and at pH values of 6.0–11.0, with an optimum of 7.0–8.0. The salinity range suitable for growth was 0–20% (w/v) NaCl, with an optimum of 12% (w/v) NaCl [20]. Strain Crm^T^ contains menaquinone MK-6 as the predominant respiratory quinone [20]. The major fatty acids (>10% of total fatty acid) are anteiso-C_15:0_ (40.61%), iso-C_15:0_ (22.0%), and anteiso-C_17:0_ (12.12%) [20]. The major cellular polar lipids are phosphatidylglycerol and diphosphatidylglycerol [20]. Glycine and lysine are the major amino acid constituents of the cell-wall hydrolysate [20].

## Genome sequencing and annotation

### Genome project history

*S. carnicancri* strain Crm^T^ was selected for sequencing because of its environmental potential as part of the Next-Generation BioGreen 21 Program (No.PJ008208). The genome project is deposited in the Genomes OnLine Database [40] and the genome sequence is deposited in GenBank. Sequencing and annotation were performed by ChunLab Inc., South Korea. A summary of the project information is shown in Table 2.

**Table 2:** Genome sequencing project information.

**MIGS ID**	**Property**	**Term**
MIGS-31	Finishing quality	Improved high-quality draft
MIGS-28	Libraries used	454 PE library (8 kb insert size) and Illumina library
MIGS-28.2	Number of reads	7,434,400 sequencing reads
MIGS-29	Sequencing platforms	454 GS FLX Titanium, Illumina Hiseq, and PacBio *RS* system
MIGS-31.2	Sequencing coverage	443.60-fold coverage (12.1 × 454 pyrosequencing, 408.4 × Illumina, and 23.1 × PacBio)
MIGS-30	Assemblers	gsAssembler 2.6, CLC Genomics Workbench 5.0
MIGS-32	Gene calling method	GLIMMER 3.02
	Genbank ID	ANAM01000000
	Genbank Date of Release	January 2, 2013
	GOLD ID	Gi21266
	NCBI project ID	175941
	Database: IMG-ER	2521172676
	Source material identifier	DSM 23852, JCM 15796, KCTC 13301
	Project relevance	Environmental and biotechnological

### Growth conditions and DNA isolation

*S. carnicancri* strain Crm^T^ was grown aerobically in marine 2216 (Marine medium, BBL), supplemented with 10% (w/v) NaCl at 30°C. Genomic DNA was extracted using a Wizard Genomic DNA Purification Kit (Promega A1120), according to the manufacturer’s instructions.

### Genome sequencing and assembly

The genome of *S. carnicancri* Crm^T^ was sequenced using a combination of a 454 Genome Sequencer FLX Titanium system (Roche Diagnostics) with an 8 kb paired end library, an Illumina Hiseq system with a 150 base pair (bp) paired end library, and a PacBio *RS* system (Pacific Biosciences). A total of 7,434,400 sequencing reads (443.6-fold genome coverage) were obtained using the Roche 454 system (187,030 reads; 12.1-fold coverage), Ilumina Hiseq system (7,219,019 reads; 408.4-fold coverage), and PacBio *RS* system (28,351 reads; 23.1-fold coverage) combined. The Roche 454 pyrosequencing and Illumina sequencing reads were assembled using Roche gsAssembler 2.6 (Roche Diagnostics) and CLCbio CLC Genomics Workbench 5.0 (CLCbio), respectively. Table 2 shows the project information and its associated MIGS version 2.0 compliance levels [27].

### Genome annotation

The open reading frames (ORFs) of the assembled genome were predicted using a combination of the Rapid Annotation using Subsystem Technology (RAST) pipeline [41] and the GLIMMER 3.02 modeling software package [42]. Comparisons of the predicted ORFs using the SEED [43], NCBI COG [44], NCBI Refseq [45], CatFam [46], Ez-Taxon-e [21], and Pfam [47] databases were conducted during gene annotation. RNAmmer 1.2 [48] and tRNAscan-SE 1.23 [49] were used to find rRNA genes and tRNA genes, respectively. Additional gene prediction analyses and functional annotation were performed using the Integrated Microbial Genomes - Expert Review (IMG-ER) platform [50].

## Genome properties

**Figure 3:**
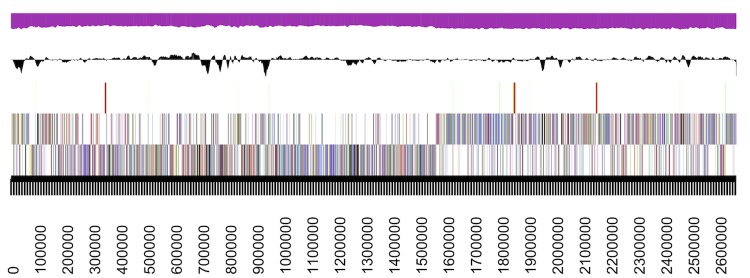
Graphical map of the largest scaffold, C792_Scaffold00001.1, which represented >99.6% of the chromosome. The smaller scaffolds of the chromosome are not shown. From bottom to top: genes on the forward strand (colored according to COG categories), genes on the reverse strand (colored according to COG categories), RNA genes (tRNAs = green, rRNAs = red, and other RNAs = black), GC content, and GC skew.

The draft genome sequence of *S. carnicancri* Crm^T^ was 2,673,309 bp, which comprised three scaffolds that included 12 contigs. The G+C content was 47.93 mol% (Figure 3 and Table 3). RAST and GLIMMER predicted 2,778 coding sequences (CDSs) in the genome. Of the predicted ORFs, 2,700 ORFs were assigned to protein-coding genes. A total of 2,298 genes (82.72%) were assigned putative functions, whereas the remaining genes were annotated as hypothetical proteins. The genome contained 78 ORFs assigned to RNA genes, including 61 predicted tRNA genes, nine rRNA genes (three 5S rRNA, three 16S rRNA, and three 23S rRNA genes), and eight other RNA genes. The distributions of genes in the COG functional categories are presented in Table 4.

**Table 3:** Genome statistics.

**Attribute**	**Value**	**% of total^a^**
Genome size (bp)	2,673,309	100.00%
DNA coding region (bp)	2,420,461	90.54%
DNA G+C content (bp)	1,279,282	47.93%
Total genes	2,778	100%
RNA genes	78	2.81%
rRNA operons	9	0.32%
Protein-coding genes	2,700	97.19%
Genes with predicted functions	2,298	82.72%
Genes in paralog clusters	1,850	66.59%
Genes assigned to COGs	2,255	81.17%
Genes assigned Pfam domains	2,333	83.98%
Genes with signal peptides	437	15.73%
Genes with transmembrane helices	679	24.44%
CRISPR repeats	1	

^a^The total is based on either the size of the genome (bp) or the total number of protein-coding genes in the annotated genome.

**Table 4:** Numbers of genes associated with the 25 general COG functional categories.

**Code**	**Value**	**%age**^a^	**Description**
J	152	5.6	Translation
A	0	0.0	RNA processing and modification
K	194	7.2	Transcription
L	120	4.4	Replication, recombination, and repair
B	1	0.0	Chromatin structure and dynamics
D	27	1.0	Cell cycle control, mitosis, and meiosis
Y	0	0.0	Nuclear structure
V	38	1.4	Defense mechanisms
T	75	2.8	Signal transduction mechanisms
M	126	4.7	Cell-wall/membrane biogenesis
N	6	0.2	Cell motility
Z	0	0.0	Cytoskeleton
W	0	0.0	Extracellular structures
U	35	1.3	Intracellular trafficking and secretion
O	71	2.6	Posttranslational modification, protein turnover, and chaperones
C	148	5.5	Energy production and conversion
G	189	7.0	Carbohydrate transport and metabolism
E	241	8.9	Amino acid transport and metabolism
F	77	2.9	Nucleotide transport and metabolism
H	120	4.4	Coenzyme transport and metabolism
I	83	3.1	Lipid transport and metabolism
P	152	5.6	Inorganic ion transport and metabolism
Q	53	2.0	Secondary metabolites biosynthesis, transport, and catabolism
R	346	12.8	General function prediction only
S	215	8.0	Function unknown
-	231	8.6	Not in COGs

^a^The total is based on the total number of protein-coding genes in the annotated genome.

## Insights from the genome sequence

*S. carnicancri* Crm^T^ encoded 72 predicted genes associated with the biosynthesis of compatible solutes and the transport of osmolytes, such as choline-glycine betaine transporter (BetT) and periplasmic glycine betaine/choline-binding lipoprotein of an ABC-type transport system (OpuBC). Potentially, these genes are key factors that allow *S. carnicancri* to adapt to high-salt environments (e.g., salt-fermented food) by regulating the osmotic concentration. Further studies are required to elucidate the osmoregulation mechanism, which could facilitate biotechnological applications of this halophilic bacterium.
